# Neural Activation during Anticipation of Near Pain-Threshold Stimulation among the Pain-Fearful

**DOI:** 10.3389/fnins.2016.00342

**Published:** 2016-07-20

**Authors:** Zhou Yang, Todd Jackson, Chengzhi Huang

**Affiliations:** ^1^Key Laboratory of Cognition and Personality, China Education Ministry, Faculty of Psychology, Southwest UniversityChongqing, China; ^2^Department of Chemistry, Southwest UniversityChongqing, China; ^3^Department of Psychology, University of MacauMacau, China

**Keywords:** fear of pain, pain anticipation, pain intensity, functional neuroimaging, midcingulate cortex

## Abstract

Fear of pain (FOP) can increase risk for chronic pain and disability but little is known about corresponding neural responses in anticipation of potential pain. In this study, more (10 women, 6 men) and less (7 women, 6 men) pain-fearful groups underwent whole-brain functional magnetic resonance imaging (fMRI) during anticipation of near pain-threshold stimulation. Groups did not differ in the proportion of stimuli judged to be painful but pain-fearful participants reported significantly more state fear prior to stimulus exposure. Within the entire sample, stronger activation was found in several pain perception regions (e.g., bilateral insula, midcingulate cortex (MCC), thalamus, superior frontal gyrus) and visual areas linked to decoding stimulus valences (inferior orbital cortex) during anticipation of “painful” stimuli. Between groups and correlation analyses indicated pain-fearful participants experienced comparatively more activity in regions implicated in evaluating potential threats and processing negative emotions during anticipation (i.e., MCC, mid occipital cortex, superior temporal pole), though group differences were not apparent in most so-called “pain matrix” regions. In sum, trait- and task-based FOP is associated with enhanced responsiveness in regions involved in threat processing and negative affect during anticipation of potentially painful stimulation.

## Introduction

The capacity to anticipate pain facilitates rapid escape and avoidance of actual and potential threats to physical integrity (Palermo et al., 2015). However, for people who experience high trait-based fear of pain (FOP) levels, such anticipation may result in excessive anxiety, fear, avoidance, and pain intensity, even when situations or actions are unlikely to cause injury (Vlaeyen and Linton, 2000, 2012). Ultimately, elevations in FOP can foster physical disuse, impairment, emotional distress, and increased pain severity within subacute, acute, and chronic pain samples (Zale et al., 2013; Jackson et al., 2014). Despite the sizable literature documenting behavior correlates and consequences of FOP, little is known about brain regions recruited in anticipation of potential pain among the highly pain-fearful. This study was conducted to elucidate this gap.

Initial research from Ochsner et al. (2006) linked higher FOP levels during the receipt of painful stimuli to stronger activation of circuits implicated in monitoring and evaluation of affective responses including the anterior and posterior cingulate cortices as well as response regulation areas (i.e., ventral lateral frontal region). These authors concluded that involvement of these regions suggests that the potential threat value of painful stimuli are more closely monitored and evaluated among the pain-fearful. In contrast, it is not clear whether the highly pain-fearful also show differential activation to cues for potential pain. Barke et al. (2012) found no activation differences between higher and lower fear of movement groups exposed to visual depictions of “aversive” vs. neutral movements. Critics of the study contended that use of visual images was insufficient in evoking differential fear responses between these groups, hence contributing to null effects for neural activation (Salomons and Davis, 2012). From this perspective, visual images that signal actual or potentially painful sensory stimulation, rather than visual images unaccompanied by such stimulation, may be more effective in evoking differential anticipatory fear and neural activation responses between higher and lower FOP groups.

Studies on general samples have found neural activity patterns corresponding to anticipation of pain overlap with those involved in pain perception. Based on a meta-analysis of 19 functional magnetic resonance imaging (fMRI) studies, Palermo et al. (2015) concluded that pain anticipation corresponds to increased activation in the anterior insula (AI), dorsolateral prefrontal cortex (DLPFC), thalamus, midcingulate cortex (MCC), medial frontal gyrus (MFG), inferior frontal gyrus (IFG) middle temporal gyrus (MTG), superior temporal gyrus (STG), inferior parietal lobule (IPL), and caudate as well as reduced anterior cingulate cortex (ACC), superior frontal gyrus (SFG), parahippocampal gyrus and claustrum activity.

Does this general activation pattern extend to anticipation of potential pain among the pain-fearful? Conceptually-related studies on anticipation of near pain-threshold stimuli have produced inconsistent results. Ploner et al. (2010) found anxious, pain-vigilant healthy adults showed weaker AI-brainstem connectivity in anticipation of near pain-threshold stimuli. Conversely, Wiech et al. (2010) reported anticipation of near pain-threshold stimulation appraised as “potentially-damaging” (i.e., fear-eliciting) predicted stronger bilateral AI activation than did anticipation of “non-damaging” stimuli. Heightened AI and MCC activity also corresponded to later judgments of near pain-threshold stimuli as painful rather than non-painful. Methodological factors may have contributed to conflicting results. The former study used trait measures of anxiety and attention to pain in male only respondents while state-based manipulations of stimulus threat values were used in the latter study. Critically, neither study directly assessed FOP. Although fear, anxiety, and threat are related, fear and anxiety may have differential effects on pain cues (Keogh et al., 2001) as well as activation patterns associated with painful stimulation (Ochsner et al., 2006). Hence, while previous near pain-threshold studies provide potentially useful foundations for hypotheses, activation patterns related to FOP are best examined using trait-related measures that tap this construct more precisely.

Toward addressing these issues, we assessed self-reported appraisals and neural responses related to trait FOP during the anticipation of potentially painful stimulation. Based on the premise that trait- and state-based FOP are correlated, we hypothesized pain-fearful participants would report more task-based fear in anticipation of stimulation and would later judge more stimuli to be painful compared to less pain-fearful controls. In addition, drawing upon Palermo et al. (2015), anticipation of stimuli later judged to be painful instead of not painful was expected to correspond to stronger activation in regions related to pain perception (e.g., AI, MCC, ACC, thalamus, DLPFC, MTG, SFG) in the entire sample. Finally, we conjectured the higher FOP group would show comparatively stronger activation in “pain perception regions” as well as those involved in the appraisal and monitoring of aversive stimuli and potential threats (Ochsner et al., 2006; Wiech et al., 2010) during stimulus anticipation.

## Experimental procedures

### Participants

The final sample included 29 emerging adults (17 women, 12 men) from a large university in Chongqing, China. Participants were 18–23 years of age (*M* = 20.28, *SD* = 1.13), right-handed, unmarried, and had 1–3 years of university education (*M* = 1.79, *SD* = 0.50). Exclusion criteria included color-blindness, presence of an ongoing medical, psychiatric or pain condition, a history of such conditions, and/or use of medication.

### Procedure

The study was approved by the university Human Research Ethics Committee. Membership within lower (*n* = 13) and higher (*n* = 16) FOP groups was based on total FOP Questionnaire-Chinese (FPQ-C) (Yang et al., 2013) scores falling, respectively, within lower and upper quartiles of the screening sample score distribution (*n* = 316), following Keogh et al. (2001). Two weeks after the screen, each willing participant attended the university's neuroimaging center for an fMRI scan. Prior to scanning, participants completed a written informed consent, demographic items, and measures of FOP and emotional distress.

Subsequently, electrodes were attached to two stimulus sites on the left foot dorsum with centers 3 cm apart from one another. Each electrode was connected to a computer controlled electrical stimulator. Stimuli were constant-current square electrical pulses of 0.5 ms duration delivered through a stainless steel concentric bipolar needle electrode consisting of a needle cathode (length: 0.1 mm, Ø: 0.2 mm) surrounded by a cylindrical anode (Ø: 1.4 mm). At painful intensities these stimuli yield pinprick-like sensations; conversely, non-painful stimuli are perceived as touch or not perceived at all.

For each participant, the near pain threshold stimulus intensity was identified and used as sensory stimulation on all study trials. Specifically, to determine individual pain thresholds, participants engaged in sensory testing at specified sites. Electrical stimulus (ES) intensities were first set at 0.2 milliamperes (mA) and increased by 0.05 mA per trial until a “just painful sensation” was indicated on a visual analog scale (VAS) with anchors of “0 = no feeling at all,” “4 = just painful sensation” and “9 = extremely painful.” Near pain threshold stimulus intensities used in the study were defined as those 0.05MA lower than one's pain threshold intensity. Pain thresholds were assessed before each fMRI run given their variability over time. Across the 4 runs, average near pain threshold stimulus intensities did not differ between higher (*M* = 1.16 mA, *SD* = 0.56; range: 0.45–2.58 mA) and lower (*M* = 1.35 mA, *SD* = 0.85; range: 0.60–3.80 mA) FOP groups, *F*_(1, 29)_ = 0.53, *p* = 0.475.

**Figure 1:**
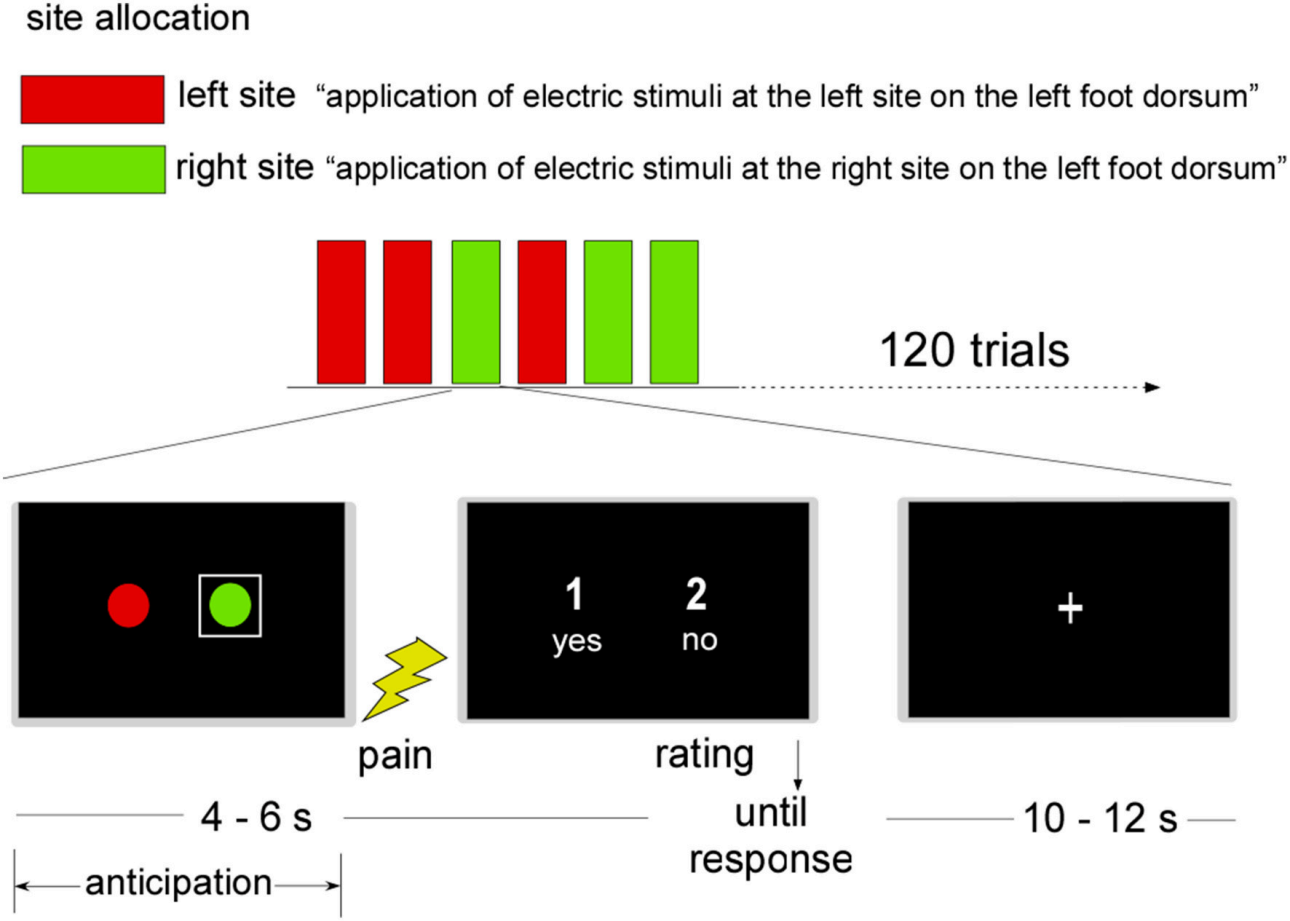
**Near-threshold pain perception task**. Note: A graphic representation of the two stimulation sites was shown before stimulus application. Stimulation sites on the left foot dorsum were represented by two circles shown on the projection screen of a 3T magnetic resonance scanner. The site stimulated in the current trial was highlighted by a white square. Subsequently, an electric stimulus, calibrated to the participant's near-threshold pain intensity, was applied. Participants were then asked to judge the stimulus as painful or non-painful (“1” = yes, “2” = no or “1” = no, “2” = yes). Following the button press, the presentation of a fixation cross ended the trial.

A judgment task adapted from Ploner et al. (2010) assessed neural responses during stimulus anticipation. Specifically, participants were told that during their scan, electrical pulses would be delivered to one of two left dorsum sites represented by a red or green circle that appeared on the scanner projection screen (see Figure 1). To signal the site of receipt on a given trial, a white square appeared around the relevant circle for 4–6 s. Whole brain fMRI scanning occurred during this phase. Next, the near-threshold pain pulse set 0.05 mA lower than the identified threshold was delivered to the cued site. Following visual cue offsets, participants judged each stimulus as painful or not painful by pressing “yes” or “no” keys within a 5 s maximum duration. After each judgment, a fixation cross (“+”) appeared for 10–12 s, followed by a new trial. To avoid sensitization or fatigue of primary nociceptive afferents, stimulus presentations were pseudo-randomized so that no more than two consecutive trials featured the same site.

The design comprised four fMRI runs of 30 trials each. Before each run, participants rated their fear levels related to the upcoming task using single item scales described below. After the fourth run, debriefing was conducted and participants were paid 60 Yuan for their time.

### Self-report measures

FOP Questionnaire-Chinese (FPQ-C; Yang et al., 2013). The FPQ-C includes 25 of the original 30 FPQ-III items (McNeil and Rainwater, 1998) based on principal components analysis and confirmatory factor analyses on Chinese adult samples. Each item was rated from “1 = not at all” to “5 = extreme.” In this study, the FPQ-C alpha was α = 0.97.

Depression, Anxiety, Stress Scale-Chinese Version (DASS-SF) (Taouk et al., 2001): The 21-item DASS-SF assessed depression (DASS-D), anxiety (DASS-A) and general stress reactions (DASS-S) experienced during the last week. Items were rated between “0 = did not apply to me at all” and “3 = applied to me much or most of the time.” The DASS-SF has acceptable reliability and validity in Chinese samples (Taouk et al., 2001). In this study, alphas were α = 0.76 (DASS-D), α = 0.69 (DASS-A), and α = 0.79 (DASS-S).

#### Task-based fear

Before each run, fear in anticipation of potentially painful sensory stimuli was assessed on VAS having anchors of “0 = no fear at all” and “100 = extreme fear.” Average scores were derived from the sum of individual ratings. The alpha for fear ratings was very high across runs: α = 0.98.

### Design and data analysis for behavior responses

A one-factor between groups design (high vs. low FOP level) featuring independent samples *t*-tests was used to assess the impact of trait FOP level on (1) intensity of fear to anticipated potentially painful stimulation and (2) the overall percentage of near pain threshold sensory stimuli judged to be painful rather than not painful.

### fMRI data acquisition and analysis

#### fMRI data acquisition

Scans were performed with a Siemens 3 T scanner (Siemens Magnetom Trio TIM, Erlangen, Germany). Each participant's head was immobilized with foam pieces during scanning to reduce motion artifacts 32 transversal slices of functional images that covered the whole brain were acquired via an echo-planar imaging (EPI) sequence. 1592 T2^*^-weighted images were collected from the 4 runs. Parameters were as follows: matrix size = 64 × 64; in-plane resolution = 3.4 × 3.4 × 3 mm^3^; TR = 2000 ms; TE = 30 ms; FoV = 220 × 220 mm^2^; flip angle = 90°; inter-slice skip = 0.99 mm. For each participant, a standard 3D T1-weighted sequence was acquired with these parameters: 176 slices at a thickness of 1 mm; in-plane resolution of 1 × 1 mm^2^; TR = 1900 ms; TE = 2.52 ms; FoV = 250 × 250 mm^2^; flip angle = 9°.

#### Pre-processing data

SPM8 (Welcome Department of Cognitive Neurology, London, UK, http://www.fil.ion.ucl.ac.uk/spm/software/spm8/) was used for image pre-processing (Friston et al., 1995). Slice timing corrected slice order and data were realigned to estimate and modify six head movement parameters. The first six images for each participant were discarded to achieve magnet-steady images normalized to MNI space in 3 × 3 × 3 mm^3^ voxel sizes. Normalized data were spatially smoothed with a Gaussian kernel; the full width at half maximum (FWHM) was specified as 8 × 8 × 8 mm^3^.

#### Whole brain activation analysis

After pre-processing and the classification of participant judgments of each near pain-threshold stimulus as painful or not painful, 2 (Stimulus Judgment) × 2 (FOP Group) analyses of whole brain fMRI data were conducted to examine neural activation differences during the stimulus anticipation phase (i.e., activation before any stimulation had been delivered and stimulus judgments had been made). Associated neural activity was assessed for differences between (1) stimuli judged to be painful vs. non-painful and (2) higher vs. lower FOP groups. Participant-specific (first-level) general linear models (GLM) included regressors for painful vs. non-painful stimulus judgments related to stimulus anticipation. Six realignment parameters for each participant were included as covariates. Button presses were included as regressors-of-no-interest. Stimulus anticipation was modeled according to duration (i.e., 4–6 s). Inferences about activation during anticipation were made at the second (i.e., between-participants) level based on contrasts between painful vs. non-painful judgments and higher vs. lower FOP group membership. Significant activity was identified at the voxel (*p* < 0.001, FDR < 0.05 corrected) and cluster levels for values exceeding a *p*-value of 0.05 (corrected for multiple comparisons).

#### Correlation analysis

Correlation analyses were conducted to examine associations between task-related fear and BOLD signal changes regions that differed between higher and lower FOP groups. First, a region-of-interest (ROI) analysis was conducted with signal intensity parameter estimates for each cluster on which FOP groups differed. Parameter estimates in spheres with a radius of 6 mm centered at peak voxels were calculated, using MarsBaR, by contrasting stimuli judged to be painful with those judged as non-painful during anticipation. Next, parameter estimates for each participant were extracted and subjected to bivariate correlations with fear of anticipated stimuli.

## Results

### Behavior results

FOP groups did not differ on demographics, depression, anxiety, or stress (Table 1). However, the higher FOP group had a significantly higher mean FPQ-C score and experienced more fear related to upcoming stimulus presentations. Contrary to predictions, FOP groups did not differ on mean percentages of stimuli judged to be painful vs. non-painful (see Table 1).

**Table 1:** **Self-report measures differences between the higher and the lower fear of pain (FOP) groups**.

**Measure**	**High FOP (*n* = 16)**	**Low FOP (*n* = 13)**	**df *t*/χ^2^**	***p***
Men/Women	6/10	6/7	χ^2^(1, 29) = 0.22	*p*>0.05
Ethnicity (Han vs. Ethnic Minority)	13/3	13/0	χ^2^(2, 29) = 2.72	*p*>0.05
Age	20.50 (1.21)	20.00 (1.00)	*t*(27) = 1.19	*p*>0.05
Years in university	1.75 (0.58)	1.85 (0.38)	*t*(27) = −0.27	*p*>0.05
FPQ-III-C	95.88 (7.92)	53.38 (8.61)	*t*(27) = 13.81	*p* < 0.001
DASS-D	3.36 (3.64)	2.18 (1.95)	*t*(27) = 1.05	*p*>0.05
DASS-A	5.03 (3.46)	3.88 (2.06)	*t*(27) = 1.06	*p*>0.05
DASS-S	5.18 (4.23)	4.88 (3.28)	*t*(27) = 0.21	*p*>0.05
Fear to the anticipated potentially painful stimuli	50.78 (17.00)	21.73 (11.34)	*t*(27) = 5.27	*p* < 0.001
Percentage of stimuli judged to be painful (%)	49.79 (20.92)	53.72 (11.77)	*t*(27) = 0.60	*p*>0.05

*FPQ-III-C, Fear of Pain Questionnaire-III-Chinese; Depression Anxiety Stress Scale, DASS*.

### fMRI results

Analyses of activation differences during stimulus anticipation resulted in significant main effects for Stimulus Judgments (painful vs. not-painful) and FOP group but their interaction was not significant (*p* > 0.05).

#### Anticipation differences in activation related to stimulus judgments

Stimuli that were later appraised as painful rather than non-painful corresponded to more activation of the left and right insula, right MCC, left thalamus, left inferior orbitofrontal cortex (OFC), right SFG, left caudate, and right pallidum during anticipation and prior to the delivery of stimulation (Table 2). In contrast, no activation differences were found during the anticipation of stimuli that were later judged to be not painful.

**Table 2:** **Activation differences during anticipation of near pain threshold stimuli later judged to be painful and non-painful within the entire sample (*N* = 29)**.

**Brain region**		**Brodmann area**	**Peak *t*-value**	**No. voxels**	**Coordinate peak**		
					***x***	***y***	***z***
**Painful** > **Non-painful**							
Insula	L	13	3.95	52	−42	3	3
Insula	R	13	4.36	69	34	13	12
Midcingulate cortex	R	32	4.27	74	3	9	39
Thalamus	L	47	3.51	8	−9	−18	0
Inferior orbitofrontal cortex	L	47	3.74	9	−36	21	−6
Superior frontal gyrus	R	6	3.94	84	−18	12	69
Caudate	L		4.46	23	−9	9	3
Pallidum	R		4.16	21	12	3	−3
**Non-painful** > **Painful: No activation differences**							

#### Anticipation differences in neural activation between FOP groups

**Figure 2:**
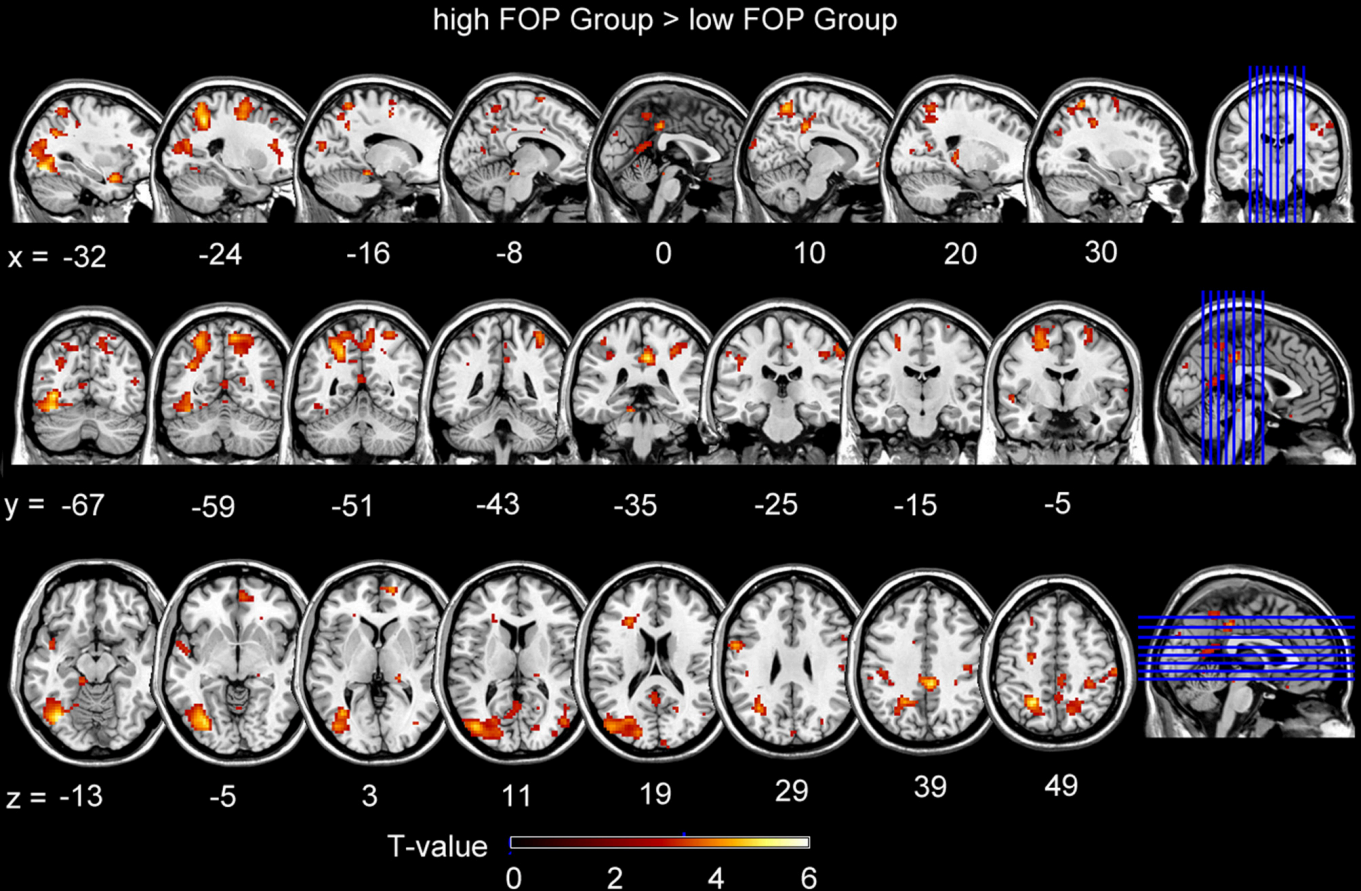
**Brain activations for contrasts involving the higher FOP Group minus the low FOP group during stimulus anticipation**. Activations in the images below were reported at a statistical threshold of *P* < 0.001, corrected by *FDR* < 0.05.

For FOP group contrasts, whole-brain analyses (*p* < 0.001, FDR < 0.05 corrected) indicated the higher FOP group showed significantly more activation during stimulus anticipation in the right MCC, left middle occipital cortex (MOC), left superior temporal pole (STP), bilateral SFG, left and right superior frontal cortices, right MTG, right supramarginal gyrus, right postcentral gyrus, and left precentral gyrus (Table 3, Figure 2). Conversely, no activation differences were found for the lower minus higher FOP group contrast.

**Table 3:** **Activation differences between higher and lower fear of pain groups in anticipation of near pain-threshold stimulation**.

**Brain region**		**Brodmann area**	**Peak *t*-value**	**No. voxels**	**Coordinate peak**		
					***x***	***y***	***z***
**Higher FOP** > **Lower FOP**							
Midcingulate cortex	R	31	5.00	57	6	−36	39
Superior frontal cortex	R	10	4.22	21	15	57	3
Superior frontal cortex	L	6	4.18	49	−24	−9	51
Middle occipital cortex/Superior	L	19/39	4.58	282	−40	−81	12
**Parietal Cortex/Inferior Occipital Cortex**							
Superior temporal pole	L	38	4.52	19	−33	6	−21
Middle temporal gyrus	R	22/39	3.91	18	48	−75	12
Supramarginal gyrus	R	40	3.85	10	60	−24	48
Postcentral gyrus	R	2	4.82	54	36	−45	63
Precentral gyrus	L	4	4.32	38	−57	3	30
**Lower FOP** > **Higher FOP: No activation difference**							

Supplementary analyses were also performed separately for each of the four fMRI runs, given the possibility that repeated exposure might result in habituation and corresponding decreases in fear and/or stimulation perceived to be painful. Results for individual runs were highly consistent with those averaged across all runs. Neither reported fear levels prior to each run nor painful stimulus judgment frequencies differed between runs. Furthermore, within the entire sample, pre-run fear levels were high correlated with one another as were painful stimulus judgment frequencies.

#### Correlations between fear of anticipated pain and brain activities in FoP group differences

**Figure 3:**
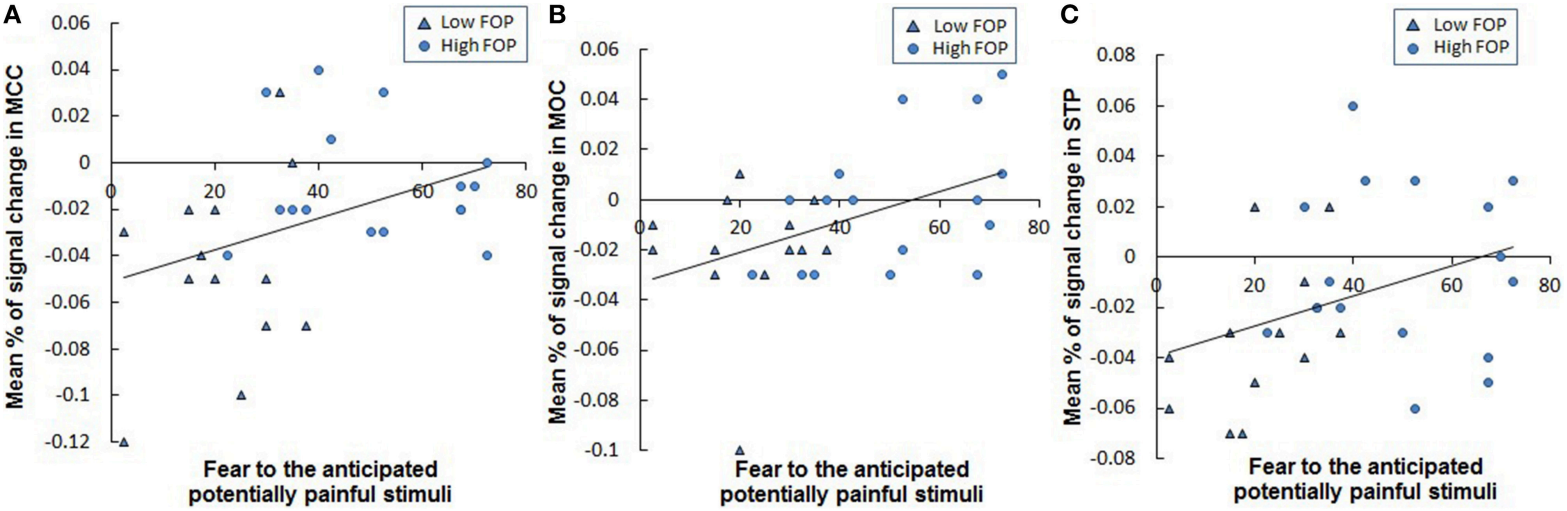
**Correlations between fear of anticipated potentially painful stimulation and the mean signal change percentages in the (A) MCC (*r* = 0.378, *p* = 0.043), (B) MOC (*r* = 0.440, *p* = 0.017), and (C) STP (*r* = 0.375, *p* = 0.058) within the whole sample**. MCC: Midcingulate Cortex; IOC: Inferior Occipital Cortex; Participants in lower vs. higher FOP Group were identified by different symbols in the above figures.

Correlation analyses indicated elevations in reported fear of anticipated stimulation were associated with stronger BOLD signal change differences in the right MCC (*r* = 0.378, *p* = 0.043; Figure 3A), left MOC (*r* = 0.459, *p* = 0.012; Figure 3B) and left STP (*r* = 0.375, *p* = 0.058; Figure 3C). In contrast, associations between task-related fear and BOLD signal change in other brain areas differentiating FOP groups (see Table 3) were not significant (all *p*'s > 0.11).

## Discussion

Despite its status as an important influence on risk for chronic pain and pain-related disability, comparatively little is known about neural responses underlying FOP. Toward addressing this gap, fMRI activation differences between higher and lower FOP groups were evaluated during anticipation of near pain-threshold stimulation. In relation to overall task perceptions, the higher FOP group experienced more task-related apprehension as reflected by significantly higher fear ratings at the prospect of receiving potentially painful stimulation. This finding aligns with research linking fear of injury to task relevant fear (Thibodeau et al., 2013).

Despite group differences in task-based fear, higher FOP group members were no more likely than controls to judge sensory stimuli as painful rather than non-painful. This finding contrasts with a recent conceptually-related meta-analysis results underscoring a modest, highly significant association between fear/threat and intensity of reported laboratory pain (Jackson et al., 2014). However, most studies from that review evaluated current or maximum pain intensities participants could bear in contrast to slightly lower than pain-threshold stimulation used in this research. Regardless, because pain judgment frequencies did not differ between FOP groups, activation differences that emerged during anticipation were more likely a function of group differences in fear than pain sensitivity.

Hypothesized FOP group differences in activation during stimulus anticipation received some support. Corresponding to group difference and correlational analyses of task-related fear, the higher FOP group showed more right MCC activation in anticipation of potentially painful stimulation. The MCC has been linked to more threatening appraisals of potentially painful stimuli during anticipation (Wiech et al., 2010), is recruited during orientation to salient, painful stimuli (Beckmann et al., 2009), and has an important role in the development of anxiety and depression (Paulus and Stein, 2010).

In addition, both between groups and correlation analyses linked higher FOP levels to stronger activation in the left MOC and left STP during anticipation of somatosensory stimulation. Several investigators have indicated increased activation in middle temporo-occipital areas corresponds to the processing of salient visual stimuli (Critchley et al., 2005; Carlson et al., 2009, 2011) as well as the generation of negative emotions (Seiferth et al., 2008; Rodríguez et al., 2015; Sepede et al., 2015). Coupled with such evidence, this activation pattern may reflect selectively increased generation and persistence of aversive affect among pain-fearful individuals confronted with the possibility of even mildly painful stimulation.

Contrary to predictions, AI activation was not related to trait or state FOP levels in the sample. This area has been implicated in the use of previous cognitive information during afferent processing of pain (Starr et al., 2009), but related studies have reported both reduced (Ploner et al., 2010) and increased AI responsiveness (Wiech et al., 2010) in anticipation of near pain-threshold stimulation. Methodological variations between the current study and past studies may have contributed to inconsistent AI results. Specifically, we evaluated trait-based FOP in a mixed sex sample while Ploner et al. (2010) examined anxiety in a male only sample and Wiech et al. (2010) used stimulus-specific manipulations of threat.

Aside from the activation differences associated with FOP, within the entire sample, anticipation of stimuli later judged to be painful rather than non-painful was related to stronger activation in several regions implicated in a recent meta-analysis of pain anticipation (Palermo et al., 2015): the bilateral insula, right MCC, left thalamus, right SFG, caudate and pallidum. In particular, enhanced insula and MCC activity during pain anticipation may reflect features of a “salience network” involved in integrating the significance of impending stimulation with perceptual decisions about pain (Atlas et al., 2010; Wiech et al., 2010; Lutz et al., 2013). Wiech et al. (2010) previously reported MCC-insula activity was reduced when participants believed that upcoming pain stimuli were entirely safe; the pattern observed in this sample highlighted reduced responsiveness of these areas prior to receiving stimulation or consciously generating judgments of stimuli as not painful. Notably, increased inferior OFC activation was also observed in anticipation of stimulation that was subsequently judged as painful. The OFC is associated with decoding stimulus valences, assessing the significance of upcoming events during anticipation of aversive stimulation (Atlas and Wager, 2012; Bolstad et al., 2013), and expectations related to changing pain experiences (Garcia-Larrea and Peyron, 2013). Other regions activated in anticipation of stimuli judged to be painful (left thalamus, left caudate, right pallidum) have also been linked to expectancies in other research (Kober et al., 2008).

Given that increased right MCC, left MOC, and left STP activation was related to both trait and state FOP during anticipation of potentially painful stimulation, this pattern may also be salient for the risk of developing chronic pain syndromes such as central sensitivity syndrome. For example, previous research has indicated that central sensitization linked to prolonged but reversible increases in central nociceptive pathway neurons can be elicited in humans by delivering noxious conditioning stimuli to skin, muscles or viscera (Woolf, 2011). Building upon this contention, results of the current study indicated that central sensitization may also be evoked by the anticipation of potentially noxious stimuli among persons who experience high state and trait FOP levels.

A key methodological strength of this study was the pairing of visual cues with subsequent somatosensory stimulation (Salomons and Davis, 2012) rather than the use of visual images in isolation of potentially painful stimulation. This strategy may have been useful in evoking differential subjective state fear responses and neural activation patterns in FOP groups. Furthermore, use of higher and lower FOP groups having no differences on demographic (i.e., gender, age, education) or common forms of general emotional distress helped to ensure any neural activation differences that emerged were attributable to FOP *per se* rather than other factors. Finally, this research extended important past work on neural correlates of FOP during pain stimulation (Ochsner et al., 2006; Ploner et al., 2010) to associations between FOP and neural responses during the anticipation of potentially painful stimulation.

Strengths aside, the main study limitations also warrant mention. First, it is not clear whether findings generalize to clinical pain samples with varying FOP levels. Use of the current paradigm in such extensions is an important future research focus. Second, use of very brief electrical pulses (0.5 ms) mitigated against assessing neural activity related to FOP during painful stimulation. While this has been a focus of key early work (e.g., Ochsner et al., 2006), extensions that consider activation during anticipation as well as receipt of prolonged somatosensory stimulation can elucidate activation patterns underlying the process of pain perception more fully.

## Conclusions

This research appears to be the first to link trait and state FOP levels with a unique pattern of neural activation in anticipation of potentially painful stimulation. In particular, during the anticipation of potentially painful stimulation, both trait-based FOP and task-based fear corresponded with elevations in the right MCC, left MOC, and left STP key regions recruited in the processing of threat and generation of negative emotions. Conversely, FOP groups did not show activation differences in areas typically involved in pain perception during stimulus anticipation and did not differ in painful vs. non-painful appraisals of somatosensory stimulus events.

## Author contributions

ZY contributed to the research design, ran the main experiment, performed all data analyses, and wrote the bulk of final Method and Results sections. TJ also contributed to conceptualization and design, and wrote the bulk of final versions of the Abstract, Introduction, and Discussion. CH was involved in formulating the research and co-supervising the first author's work on the project.

## Funding

This research was supported by the Post-Doctoral Foundation of Chongqing (Xm2015102) to the first author and grants from the National Natural Science Foundation of China (31371037), Education Ministry of China, and Chongqing 100 Persons Fellowship to the corresponding author.

### Conflict of interest statement

The authors declare that the research was conducted in the absence of any commercial or financial relationships that could be construed as a potential conflict of interest.
